# Forced residential mobility and social support: impacts on psychiatric disorders among Somali migrants

**DOI:** 10.1186/1472-698X-12-4

**Published:** 2012-04-17

**Authors:** Kamaldeep Bhui, Salaad Mohamud, Nasir Warfa, Sarah Curtis, Stephen Stansfeld, Tom Craig

**Affiliations:** 1 Wolfson Institute of Preventive Medicine, Barts & The London School of Medicine & Dentistry, Queen Mary, University of London, London, UK; 2 University of Durham, Durham,UK; 3 Institute of Psychiatry, King's, College London, London, UK

## Abstract

**Background:**

Somali migrants fleeing the civil war in their country face punishing journeys, the loss of homes, possessions, and bereavement. On arrival in the host country they encounter poverty, hostility, and residential instability which may also undermine their mental health.

**Methods:**

An in-depth and semi-structured interview was used to gather detailed accommodation histories for a five year period from 142 Somali migrants recruited in community venues and primary care. Post-codes were verified and geo-mapped to calculate characteristics of residential location including deprivation indices, the number of moves and the distances between residential moves. We asked about the reasons for changing accommodation, perceived discrimination, asylum status, traumatic experiences, social support, employment and demographic factors. These factors were assessed alongside characteristics of residential mobility as correlates of ICD-10 psychiatric disorders.

**Results:**

Those who were forced to move homes were more likely to have an ICD-10 psychiatric disorder (OR = 2.64, 1.16-5.98, p = 0.02) compared with those moving through their own choice. A lower risk of psychiatric disorders was found for people with larger friendship networks (0.35, 0.14-0.84, p = 0.02), for those with more confiding emotional support (0.42, 0.18-1.0, p = 0.05), and for those who had not moved during the study period (OR = 0.21, 0.07-0.62, p = 0.01).

**Conclusions:**

Forced residential mobility is a risk factor for psychiatric disorder; social support may contribute to resilience against psychiatric disorders associated with residential mobility.

## Background

Migration can be conceptualized as arising due to 'push' factors like civil war, persecution, and discrimination; these are also determinants of psychiatric disorders [1]. Migration can also occur due to 'pull' factors like seeking employment, and to live with friends and family. Asylum seekers and refugees are at greater risk of psychiatric disorders because of the risks associated with migration itself [1], and due to severe traumatic life events, usually in the context of conflict and persecution, before and during asylum journeys [2]. Contrasting with the hazards before migration and during asylum-seeking journeys, some research is beginning to show that adversities in the country providing asylum may be the more important determinants of psychiatric illness [3,4].

The majority of asylum seekers and refugee migrants in the UK are living in the capital city, London [5], where there is poor quality housing, significant material and area deprivation which contribute to higher risks of psychiatric disorders [6]. Asylum seekers may be detained at the port of entry whilst their documents are scrutinized; they may be subjected to interrogation about seeking illegal entry; they may be declined asylum and refugee status on the basis of inconsistent stories even though it is known poor recall occurs more commonly amongst traumatized refugee populations [7]. While their asylum application is processed they may be held in detention centres for long periods of time or in public housing provided by the authorities. Ultimately, even if they are granted refugee status, they may be re-settled in a city or town which is not of their own choosing, and it may be a long way from friends, relatives, and away from people in whom they can confide and whom they trust [8]. Stigma and discrimination, known to be associated with psychiatric disorders, is a particularly common experience if the host population perceives asylum seekers and refugees are favoured in receiving jobs and housing. Furthermore, some countries (the UK for example, unlike the US) prohibit employment whilst the asylum application is considered, and do not offer more than minimal financial support whilst awaiting refugee status. People with psychiatric disorders also tend to descend the social ladder, a process called social drift, and end up living in impoverished housing and in poorer neighborhoods as their illness prevents them from securing income and permanent housing.

Although a great deal of literature considers the impact of international migration on psychiatric disorders, especially in asylum seekers and refugee populations, there is little that examines local in-country migration as a determinant of psychiatric disorders. Little has been done to test whether local residential movement is equally or more important than other adverse experiences. This paper presents new findings about local residential mobility and psychiatric disorders among Somali migrants (asylum seekers and refugees) living in East and South London. The study made use of an innovative method to describe different patterns of residential mobility. The frequency and distance travelled during residential moves, the relative deprivation of the neighborhoods between moves, and the role of personal choice (versus forced moves) over changes in accommodation are specifically investigated as risks for psychiatric disorders whilst also taking into account social support, employment, and pre- and post-migration social adversity (discrimination, traumatic events). The majority of refugee migrants do not have psychiatric disorders. So this study also investigates supportive relationships with friends, families and wider networks which are known to offer some protections against emotional problems and ill health in general. Can social support buffer against psychiatric disorders related to residential mobility?

Specifically, the study hypotheses are that

1 Psychiatric disorders are more common among those showing residential movements.

2 Choice over the residential move is associated with a lower prevalence of psychiatric disorders.

3 Social support mitigates the risk of psychiatric disorders, whilst discrimination and traumatic experience increase the risks of psychiatric disorders.

## Methods

### Sample & recruitment

The study was carried out between May 2001 and August 2003, in the London Boroughs of Tower Hamlets (population 196,000) and Lambeth (population 266,800). Refugees numbered approximately 26-29 per 1000 residents in Tower Hamlets and 35-41 per 1000 residents in Lambeth [9]. These boroughs are amongst the most deprived in London (Townsend Index^a ^score of 13 and 10 for the year 2001; range from +13 = most deprived, to 1 = least deprived).

The sampling methods have previously been reported, but briefly, the approach aimed to recruit as close to a population sample as possible in the absence of an enumerated sampling frame for Somali people [9,10] A mixed-method of sampling was used. One sample source was a random sample of primary care patients who were registered with Somali names; these subjects were verified to be of Somali origin by a Somali speaking researcher who contacted the individual by phone, letter or house call. Also, preceding the survey, Somali researchers (NW, SM) with the necessary linguistic skills and cultural knowledge networked with local stakeholders, and undertook focus groups to improve engagement, pilot methods to undertake the study, and identify suitable community sampling venues [11]. Six focus groups (2 with local Somali professionals; 4 with Somali lay people) identified the community venues from which we recruited Somalis for the survey: Somali cafes, community centres, mosques, further education colleges and refugee hostels. We were assured by focus group participants that these venues were equally likely as a whole to yield samples of men and women, and that these places were not directly linked to services for specific health conditions. During the main survey, every Somali entering any of these community sites was approached for screening, and either interviewed there or an interview appointment was arranged. The next Somali to enter the site when the researcher was next free was then approached for screening and the procedure was repeated. We aimed to recruit 150 participants (men and women) between the ages of 18 and 65. Of these 50 were to be recruited from general practice registers (GP's) and the remaining 100 recruited from the non-conventional places such as Somali cafés, community centres, mosques, further education (FE) colleges and refugees hostels. The power calculations showed this sample size to be sufficient to show a prevalence difference of at least 25% between two comparison groups (assuming 40% of refugees had a psychiatric disorder [12], with a power of 90% to a significance level of 5%.

Subjects were eligible for a research interview if they were of Somali origin, gave informed consent and were resident in the London Boroughs of Tower Hamlets (East London) or Lambeth (South London) at the time of the survey. Somali origin was defined as having migrated from Somalia to the UK by any route for resettlement in the preceding 5 1/2 years, and being of Black African ethnic group. We wished to recruit recent migrants who were more likely to show significant residential instability. Focus groups recommended that Somali migrants became residentially stable after about 5 years.^b ^Full appropriate Institutional Review Board ethical approval was obtained from Local Health Authorities in both sites. Participants, who completed the interview, received a nominal expenses payment of £10 (ten UK pounds).

### Survey questionnaire, data management and analysis

The survey questions asked about age, gender, legal status (asylum seeker or refugee status granted), employment status, and types of welfare benefits being received.

### Residential mobility

The *accommodation record questionnaire *and methods of gathering information about residential mobility were adopted from a study of homelessness in young people [13]. This questionnaire records every place of residence of two weeks or more at any particular address. Information is gathered about tenure status (permanent or temporary) and balance of factors determining the decision to move (in particular the extent to which the move was the participant's own choice). Participants were asked to come to the research interview with written address information, or at least information on the first line of their address, including a postcode, or, if they were new in the country, roughly the area they have lived and memorable local landmarks; for example, a nearby popular place such as a stadium, cinema, a market etc. from which a good approximation of the post code could be derived. These details were repeated for each change of address over the preceding 5.5 years. In order to facilitate recall, key time points (birthdays, memorable events etc.) in their life were charted and used to aid discussion of residential location across the period of interest.

The 6 or 7 alphanumeric post code provided the basis for locating residence to small geographic units. Some participants did not know the exact post-codes of the previous addresses and in order to overcome this problem, we used a number of strategies. We searched the online databases such as official Royal Mail post-code finder where street name and house number was known and wider mapping tools to identify locations of streets where this was the only information. We also hand searched the postal address book for London (2001 edition). This was particularly useful for those who provided only street names or incomplete post-codes. Ambiguous addresses were clarified by follow up visits or telephone calls.

A total of 342 addresses were assigned either a full postcodes or partial codes based on all the information provided. Fifty nine addresses were incomplete or missing, thus 85% of all potential postcodes were available for the analysis. Overall, 137 (95.8%) had adequate information to assign a postcode for their address at the time of the survey; 94 (87%) of participants who moved at least once and therefore had a second address could be assigned a post-code; post codes could be assigned to between 62% to 100% of those with 3 or more changes of accommodation.

**Figure 1:**
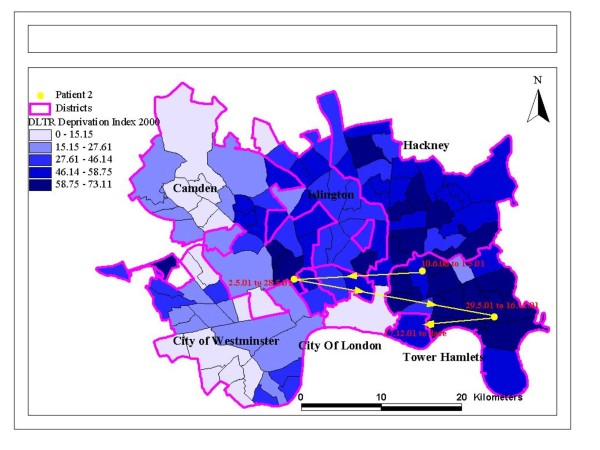
**Mapping mobility between different levels of area deprivation**.

**Figure 2:**
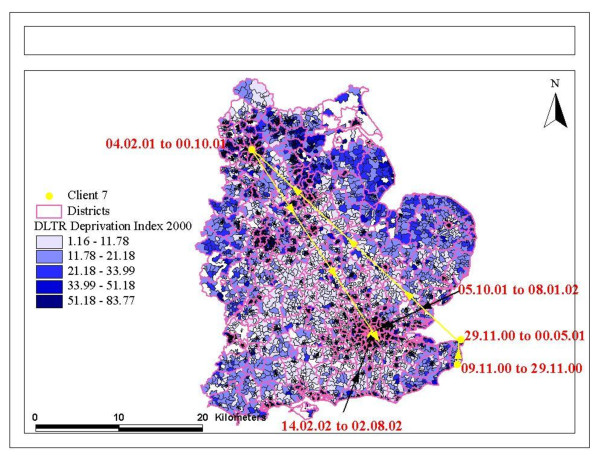
**Mapping mobility between different levels of area deprivation**.

Post-codes were then linked to respective electoral wards (as defined in 1998) and a publically available *Index of Multiple Deprivation *(IMD) that was based on housing, education, health and child deprivation (http://data.gov.uk/dataset/index-of-multiple-deprivation). Post-codes were also used in a digitised grid reference locator. The generated file provided data on the distances moved between post-codes and therefore we were able to calculate distances between residential moves, and to map moves between specific high and low deprivation areas (see Figure 1 &2 showing dates, and movements by deprivation of areas).

### Measures of psychiatric disorders and health status

The Mini Neuropsychiatric Interview (MINI) assigns a DSMIV and ICD-10 diagnoses of psychiatric disorders (depression, mania, panic disorder, post traumatic stress disorder, alcohol and drug dependence, psychosis, anxiety and anti-social personality disorder and suicidal risk)[14]. The instrument is highly structured and can be administered by non-clinicians producing a valid and reliable diagnostic assessment. The MINI diagnostic framework allows for multiple psychiatric disorders, and distinguishes current psychiatric disorders from those arising at an earlier stage of the subject's life. It is currently available in over 40 languages.

The MINI and the other instruments were carefully forward and back translated, using consensus panels; with tests of reliability and validity that showed these to perform well; details of the cultural adaptation of measures and indices of reliability and validity are separately published along with the training of lay interviewers [9]. For this analysis, the category of *any psychiatric disorder *was used as an outcome in regression models which tested whether the characteristics of residential mobility measured were independent risks factor for psychiatric disorder. The MINI includes a question about experiencing or witnessing something very traumatic. A yes answer to this screening question, as a measure of *previous trauma exposure*, a *PTSD *diagnosis, and any psychiatric disorder (ICD-10 and DSMIV diagnoses including PTSD) were used in descriptive analyses to test associations with residential mobility. General health was assessed using the first question of the Short Form-12 [15], to indicate those reporting poor or fair health and comparing with those reported, good, very good or excellent health.

### Social support

Social support was assessed by an adapted version of the *Close Person's Questionnaire *[16]. Participants were asked to rate how much in the 'last 12 months did this person make you feel good about yourself; shared interests, hobbies or had fun; did you confide in that person.' Each of these three domains were scored as 0, 1 or 2 (0: not at all, 1: a little. 2: quite a lot/a great deal). The total score (0 to 6) and a binary variable (derived by applying the median score as the threshold) were used in analyses.

Within the questionnaire about social support, we also asked about the size of the friendship network and relatives' network; we asked if there were any relatives (and friends in a separate question) (yes = 1, no = 0), frequency of contact (almost daily = 4, once a week, once a month, once every few months, never/almost never = 0), frequency of visits (almost daily = 4, once a week, once a month, once every few months, never/almost never = 0); how many relatives (and friends in a separate question) were seen at least once a month (6 or more = 3, 3-5, 1-2, none = 0). The total score for relatives (0-12) and the total score for friends (0-12) were used in analysis. A higher score indicates a larger network.

### Discrimination

Questions from an earlier national study (EMPIRIC) were used to measure experiences of discrimination [17,18]. These explored experiences and events in the preceding 12 months: physical attack, damage to property, or insults to do with ethnicity or religion or race; unfair treatment at work, or job refusal. These instances were summed to give a total discrimination events score (0-5).

### Piloting the questionnaire

Before the actual study, and after the translation process, we interviewed 18 subjects. These subjects were recruited from non-health community venues such as cafes, community centres and refugee training facilities in the two study areas. The aims of this preliminary work were to assess the ease and comprehensibility of the new instrument by the target population. The subjects were specifically asked to indicate if they did not understand some words in the sentence rather than respond to the overall meaning of the question. None of these subjects reported any problems in understanding the questions and what it referred to, suggesting that the final draft to be understandable, and conceptually appropriate.

### Statistical analyses

Descriptive analyses are presented with chi-squared statistical tests for associations with the frequency of geographical mobility (none, 1-2, 3 or more). Associations of health variables (SF12, PTSD/trauma experiences, any psychiatric disorder) with the frequency of residential mobility (none, 1-2, 3 or more; binary: none, 1 or more) are also presented with descriptive statistics. Associations with psychiatric disorder (any ICD-10 or DSM-IV diagnostic group) are then tabulated showing odds rations and 95% confidence intervals and a p value for univariate associations.

Stepwise models were built with three characteristics of residential mobility as the explanatory factor: any mobility (frequency did not show a dose response relationship), distance moved, and choice over mobility. These could not be entered into a single model due to collinearity. Stepwise models were built by forward selection and entry of variables showing univariate associations with psychiatric disorder to a significance of p < or = 0.2. The stepwise models were made more efficient by using binary variables for social support, using median scores as threshold scores. The reference group was any residential mobility (as this was the largest group) showing the protective effects of not moving. The results tables show only the retained variables, and the findings as odds ratios, 95% confidence intervals and a p value.

### Consent & ethical procedures

Consent was sought verbally and in written form, and where there were literacy problems, verbal consent sufficed but had to be countersigned by the investigator. The procedures were approved by the ethics committees, and included leaving an information leaflet with subjects should they wish to seek advice or information at a later date. Preceding the survey, Somali researchers (NW, SM) with the necessary linguistic skills and cultural knowledge networked with local stakeholders. The researchers encouraged trust by attendance at sampling venues and Somali cultural events where they provided information about the study. They also explored what facilitated and what hindered engagement in research using the focus groups in the preliminary work. The work was undertaken flexibly at weekends and evenings, as well as during regular working hours to maximize participation for a hard-to-reach population. Full appropriate Institutional Review Board ethical approval was obtained from Local Health Authorities in both sites.

## Results

### Correlates of residentially mobility

Choice over the move was not associated with housing status (temporary or permanent) at the time of the study or at the previous address (not significant, data not reported).

The maps of residential mobility show moves within a local area, and moves between areas with different deprivation indices (Figure 1 &2). These show that movements appear not to be related to deprivation, and can cross administrative boundaries. This is confirmed by statistical tests shown in Table 1. Other descriptive correlates of residential mobility are presented in Table 1. Women, residency in the UK for over 2 years, people on job seekers allowance or on income support, and those exposed to discrimination were more likely to move more frequently. Employment was related to frequency of moves, with most employed people having moved 1-2 times, and fewer having no moves or moved on 3 or more occasions. The distance moved did not relate to frequency of moves.

**Table 1:** Frequency of residential mobility: demographic, social, environpsychiatric, legal & health influences

Variables (N)	Categories	No Move (%)	1-2 Moves (%)	3-6 Moves (%)	*χ*^2^	df	P
Age groups (143)	18 - 25	14 (40)	17 (23)	8 (23.5)	5.4	4	0.2
	26 - 35	13 (37.1)	28 (37.8)	16 (47.1)			
	≥ 36	8 (22.9)	29 (39.2)	10 (29.4)			

Gender (143)	Male	**23 (65.7)**	**35 (47.3)**	**13 (38.2)**	**5.5**	**2**	**0.06**
	Female	**12 (34.3)**	**39 (52.7)**	**21 (61.8)**			

Marital	Single	17 (48.57)	37 (50)	18 (52.94)	2.72	4	0.61
	Married	17(48.57)	29 (39.19)	14 (41.18)			
	Separated/widowed/divorced	1 (2.86)	8 (10.81)	2 (5.88)			

Employment in UK (143)	Unemployed	32 (91.4)	68 (91.9)	28 (82.4)	2.4	2	0.3
	Employed	3 (8.6)	6 (8.1)	6 (17.6)			

Income support (143)	No	**28 (80.0)**	**54 (73.0)**	**17 (50.0)**	**8.3**	**2**	**0.02**
	Yes	**7 (20.0)**	**20 (27.0)**	**17 (50.0)**			

Job seekers Allowance (143)	No	**26 (74.3)**	**35 (47.3)**	**24 (70.6)**	**9.4**	**2**	**0.009**
	Yes	**9 (25.7)**	**39 (52.7)**	**10 (29.4)**			

Period of stay in the UK (143)	< 2 years	**26 (74.3)**	**35 (47.3)**	**9 (26.5)**	**16**	**2**	**0.0001**
	≥ 2 years	**9 (25.7)**	**39 (52.7)**	**25 (73.5)**			

Tenure current (142)	Temporary	**29 (85.3)**	**50 (67.6)**	**19 (55.9)**	**7**	**2**	**0.030**
	Permanent	**5 (14.7)**	**24 (32.4)**	**15 (44.1)**			

Legal status (143)	Pending	10 (28.6)	11 (14.9)	4 (11.8)	4.1	2	0.1
	Resolved	25 (71.4)	63 (85.1)	30 (88.2)			

Discrimination Score (N = 143)	Mean (sd)	**0.8(0.87)**	**1.05(1.0)**	**1.59 (0.96)**	**13.26**	**2**	**0.001**

Emotional support (143)	Mean (sd)	4.62 (1.8)	4.89(1.7)	5.38(1.37)	4.55	2	0.1

Friends network (143)	Mean(sd)	7.51(3.89)	7.92(3.4)	8.74(2.64)	1.34	2	0.51

Relatives network (143)	Mean(sd)	**6.46(3.31)**	**6.41(2.93)**	**7.97(2.71)**	**8.23**	**2**	**0.02**

Deprivation score at current address (143)	Mean (sd)	51.21(15.14)	51.37(17.02)	56.87(15.75)	3.08	2	0.22

Deprivation score at previous address (94)	Mean (sd)	0	46.6(16.84)	46.86(20.77)	0.09	1	0.76

Relative deprivation of previous and current area of residence	High to low	-	22 (33.85)	9 (31.03)	1.40	2	0.50
	Low to high	-	38 (58.46)	17 (58.62)			
	same	-	5 (7.69)	3 (10.34)			

### Correlates of psychiatric disorders

The mobility variable was coded to binary variable (no move/move)(Tables 2 &3). There were statistically significant associations of any mobility (no move/move) with general health (*X*^2 ^= 4.86, df = 1. p = 0.03), trauma history (*X*^2 ^= 4.31, df=, p = 0.04) and any psychiatric diagnosis (*X*^2 ^= 4.38, df = 1. p = 0.04), but not with PTSD (*X*^2 ^= 1.13, df = 1, p = 0.29). Trauma history was not associated with a psychiatric disorder (OR = 1.8, 95%CI: 0.55-2.52, p = 0.67). People with fair or poor self reported general health (on the SF12) were more likely to have a psychiatric disorder than those reporting excellent, good or very good general health (OR = 1.94, 95%CI: 1.41-2.67, p < 0.001).

**Table 2:** Demographic, social, legal characteristics of those with and without any psychiatric disorder meeting ICD-10 or DSM-IV diagnostic criteria

Variables (N)	Categories	No Psychiatric Disorder	Any Psychiatric Disorder	OR	95%CI	P
Age group (143)	18 - 25	27 (30.3)	12 (22.2)	1	0.6-3.36	0.43
	26-35	35 (39.3)	22 (40.7)	1.41	0.68-4.07	0.26
	35 - 65	27 (30.3)	20 (37.0)	1.67		

Gender (143)	Male	41 (46.1)	30 (55.6)	1	0.35-1.35	0.27
	Female	48 (53.9)	24 (44.4)	0.68		

Marital	Single	46 (51.69)	26 (48.14)	1	0.58-2.39	0.65
	Married	36 (40.45)	24 (44.44)	1.18	0.27-3.78	0.99
	Separated/widowed/divorced	7 (7.87)	4 (7.41)	1.01		

Employment in the UK (143)	Unemployed	76 (85.4)	52 (96.3)	1	0.05-1.04	0.06
	Employed	13 (14.6)	2 (3.7)	0.23		

Income support (N = 143)	No	61(68.54)	38(70.37)	1	0.44-1.92	0.82
	Yes	28(31.46)	16(29.63)	0.92		

Job seekers allowance (N = 143)	No	55(61.8)	30(55.56)	1	0.65-2.57	0.46
	Yes	34(38.2)	24(44.44)	1.29		

Period in the UK (143)	< 2 years	42 (47.2)	28 (51.9)	1	0.42-1.63	0.59
	≥ 2 years	47 (52.8)	26 (48.1)	0.83		

Tenure (142)	Temporary	56 (63.6)	42 (77.8)	1	0.23-1.09	0.08
	Permanent	32 (36.4)	12 (22.2)	0.5		

Legal status (143)	Pending	14 (15.7)	11 (20.4)	1	0.30-1.75	0.48
	Resolved	75 (84.3)	43 (79.6)	0.73		

Discrimination (N = 143)	Mean (sd)	1.1(0.98)	1.15(1.04)	1	0.75-1.47	0.78
				1.05		

Emotional support (143)	mean (sd)	**5.2(1.46)**	**4.52(1.94)**	**1**	**0.64-0.97**	**0.02**
				**0.78**		

Friends Network (143)	mean(sd)	**8.76(3.09)**	**6.78(3.48)**	**1**	**0.75-0.93**	**0.001**
				**0.84**		

Relatives Network (143)	Mean (sd)	**7.7(3.05)**	**5.7(3.05)**	**1**	**0.73-0.93**	**0.001**
				**0.82**		

**Table 3:** Mobility characteristics and psychiatric disorders

Variables (N)	Categories	No Psychiatric Disorder	Any Psychiatric Disorder	OR	95%CI	P
Deprivation Score at current address (N = 143)	Mean(sd)	53.7(16)	50.69 (16.89)	1	0.97-1.0	0.3
				0.99		

Personal choice to move	Not moved	**26(28.89)**	**8(14.81)**	**1**	**0.4-3.67**	**0.73**
	Own choice	**24(27.59)**	**9(16.67)**	**1.22**	**1.3-8.11**	**0.01**
	Others choice	**37(42.53)**	**37 (68.52)**	**3.25**		

Personal choice to move	Not moved/own choice	**50(57.47)**	**17(31.4)**	**1**	**1.44-6.0**	**0.003**
	Others choice	**37(42.53)**	**37 (68.52)**	**2.94**		

Total distance moved (129)	Not moved	**27 (33.8)**	**8 (16.3)**	**1**	**0.69-4.96**	**0.22**
	< 10.2 km	**31 (38.8)**	**17 (34.7)**	**1.85**	**1.38-9.79**	**0.009**
	≥ 10.2 km	**22 (27.5)**	**24 (49.0)**	**3.68**		

Total distance moved (94)	< 10.2 km	31 (38.8)	17 (34.7)	1	0.87-4.55	0.1
	≥ 10.2 km	22 (27.5)	24 (49.0)	1.99		

Distance moved in most recent move (N = 94)	Not moved	8 (15.09)	2 (4.88)	1	0.42-12.04	0.35
	<4 km	25(47.17)	14(34.15)	2.24	0.95-26.23	0.06
	>4 km	20(37.74)	25(60.98)	5		

Distance moved in most recent move (N = 84)	<4 km	25(47.17)	14(34.15)	1	0.93-5.38	0.07
	>4 km	20(37.74)	25(60.98)	2.23		

Relative deprivation of previous and current residential areas (94)	High to Low	15 (28.3)	16 (39.02)	1	0.09-2.51	0.37
	No change	6 (11.32)	2 (4.88)	0.46	0.61-3.6	0.38
	Low to High	32 (60.38)	23 (56.1)	1.48		

### Residential mobility and psychiatric disorders: Multivariate models

Psychiatric disorders were less likely among those who had not moved during the preceding five years (OR = 0.21, 0.07-0.62, p = 0.01), but were not associated with the frequency of residential moves (Table 2, 3, 4). Not having choice over the move is the equivalent of being forced to move. Forced moves occurred in 69% (74/107) people. Compared with *non-movers*, those experiencing a *forced move *were more likely to have a psychiatric disorder even after adjusting for gender and tenure (OR = 3.32, 95%CI: 1.27-8.67, p = 0.02). Compared with those *moving on a voluntary basis*, those experiencing a *forced move *remained at higher risk of a psychiatric disorder, even after adjusting for gender and tenure (OR = 2.86, 95%CI: 1.14-7.2, p = 0.03)

**Table 4:** Associations with psychiatric disorder from stepwise logistic regression models: only retained variables shown

Model 1		OR	95%CI	p value	N	R^2^
**Moved in last 5 years**	Yes	1				

**Gender**	No	0.21	0.07-0.62	0.01	128	0.21
	
	Men	1				
	
	Women	0.35	0.14-0.86	0.02		

**Friendship network**	Low	1				

	High	0.35	0.14-0.84	0.02		

**Relative network**	Low	1				

	High	0.46	0.2-1.07	0.07		

**Confiding relationships**	Low	1				

	High	0.42	0.18-1.0	0.05		

**Physical health sf12**	Poor/fair	1				

	Good/excellent	0.48	0.18-1.32	0.15		

**Employment in UK**	No	1				

	Yes	0.21	0.04-1.23	0.08		

**Model 2**						

**Choice over move**	Own choice or not moved	1			137	0.2

**Gender**	Other's choice	2.64	1.16-5.98	0.02		
	
	Men	1				
	
	Women	0.5	0.22-1.16	0.11		

**Friendship network**	Low	1				

	High	0.34	0.15-0.79	0.01		

**Relative network**	Low	1				

	High	0.48	0.21-1.09	0.08		

**Confiding relationships**	Low	1				

	High	0.43	0.19-0.99	0.05		

**Physical health sf12**	Poor/fair	1				

	Good/excellent	0.41	0.16-1.05	0.06		

**On job seekers allowance**	No	1				

	Yes	1.97	0.84-4.61	0.12		

Stepwise logistic regression models; psychiatric disorder as an outcome. Modelled by forward entry if significant to a p < 0.2 level. Variables entered including age, gender, discrimination score, networks as binary variables using median split (relatives, friendship and confiding and emotional relationships), physical health using median split on the SF12, employment in the UK (yes/no), on benefits (job seekers' allowance, or income support, marital state (single, married, sep/widowed/divorced), legal status (asylum seeker/refugee status), tenure (temporary/permanent), period living in the UK, index of multiple deprivation of the current address and trauma history (yes/no).

The distance moved both in the most recent move and in all moves in the study period was associated with a greater risk of psychiatric disorder, but this finding was not sustained in multivariate models. Confiding emotional relationships, friendship networks, and to a lesser extent, relatives networks were associated with lower risks of psychiatric disorder in multivariate models (Table 4).

## Discussion

### Main findings

Migration is known to be associated with poorer health for a number of reasons: risk-behaviors in the country of origin, new risk behaviours or loss of protective behaviours in the new country, an adverse social environment, and interactions between these [19,1]. The quality (hostile or supportive) of the social environment, national policies and attitudes to migration, and availability of social and health services all make a difference to levels of illness and resiliency[20].

Few studies have looked at local residential movements as a risk factor for health. Local geographical mobility is common in inner city areas, [11-13] where up to a third of residents have been estimated to move accommodation each year. Therefore, although there are specific risks to which refugees are exposed, the findings may be relevant to local geographical mobility more generally. We demonstrated that psychiatric disorders are more common among Somali migrants who have moved accommodation within five years of arrival in the UK, but there is not a relationship with frequency of residential moves or distance of moves. Social support networks did seem to protect against psychiatric disorder related to mobility, supporting the idea that social networks promote resilience in vulnerable populations. (see Table 4) [21]. This association may reflect successful local mobility in order to be closer to friends and relatives, by those who are less likely to be suffering from mental distress. That is, those with mental disorders may have fewer close friends and be less likely to move successfully, or with planning and choice, especially if they are in supported public housing. Or their behaviour and the stigma associated with both refugee status and mental disorders lead to frequent moves in order to avoid hostility.

Previous moves of accommodation were more common among those with permanent tenancies at the time of the survey, perhaps reflecting successful moves towards permanency. These findings suggest that not all residential mobility is motivated by forced resettlement and that elective moves account for most of the residential mobility. These findings suggest that residential mobility takes place in order to seek out social networks but also to further employment prospects.

In multivariate models, only choice over the move and social support and networks appeared to be influential. The findings suggest that the stereotypical discourse about the causes of residential mobility among refugee migrants being due to pre-migration traumas and a tendency to remain highly mobile, do not reflect Somali migrants as active agents in decisions about their future; the discourses about refugee resettlements do not reflect individual choice and social networks as influences on mobility or on risks of psychiatric disorder.

We did not show that PTSD or traumatic experiences in the past were linked with psychiatric disorders, although trauma experienced before migration was marginally associated with a greater propensity to be residentially mobile. Trauma is therefore not inevitably linked with psychiatric disorders more generally, and residential mobility need not be associated with high risks of psychiatric disorder for those in good support networks and where choice of the move is maximized. These findings argue for more agency and choice among refugees when making decisions about re-location, especially if by the authorities. Re-location to areas where relatives or friends are already living seems preferable, rather than to more isolated rural communities where fewer people from black and minority ethnic groups live, and where refugees are even more conspicuous as immigrants compared with inner city areas. Figures 1 and 2 show residential mobility can take place between areas with different levels of deprivation, and also that some people who are re-located far from an urban area in which they prefer to live, may well relocate themselves back to that area. Again, permitting maximum choice within the parameters of what is permitted by asylum and refugee legislation is important.

### Strengths and limitations

We were not able to run complex multi-level models or test for many interactions given the sample size. Twelve women did not move during the study period; the role of gender, warrants some attention, but given our small sample size we were not able to test for interactions with gender. The sample size was limited despite intensive recruitment methods that aimed to secure as close to a population sample as is possible, using previously well tested methods, and a sample with significant levels of residential mobility. Recruitments of migrants to research studies is known to be challenging, hence, these methods were important to ensure engagement and active recruitment. We analysed distance data from the most recent move and the total distance moved for up to five years since arrival, both giving the same picture, that is a marginally significant results with greater distances being associated with greater psychiatric disorders. This provides some validation of the methods, given the most recent residential move is likely to be the best recalled. The study was not undertaken prospectively, but relied on an innovative interview technique using time related anchor points generated by the interviewee to ensure their recall is maximized. This has previously been used successfully with homeless youth and worked well in this study where we identified a significant proportion of addresses using this information.

## Conclusions

Forced residential mobility is a risk factor for psychiatric disorder; social support may contribute to resilience against psychiatric disorders associated with residential mobility. Re-location to areas where relatives or friends live is preferable and healthier. Permitting maximum choice of residence within the parameters of what is permitted by asylum and refugee legislation is important.

## Endnotes

^a^The four variables that comprise the Townsend Index are a) unemployment as a percentage of those aged 16 and over who are economically active; b) non-car ownership, as a percentage of all households; c) non-home ownership as a percentage of all households; d) household overcrowding. The four variables combine to form an overall score. The higher the Townsend Index score, the more deprived and disadvantaged an area is thought to be. This allows different areas to be ranked in relation to one another.

^b^In order not to exclude residents who had lived in the UK for a few days or months more than a 5 year period, we included those who reported UK residence for up to 5.5 years. We excluded subjects who lived outside the two London boroughs at the time of the first interview, and those we could not follow up because of incorrect address or contact information, or if they were transferred out of temporary accommodation before agreeing an interview.

http://www.warwickshire.gov.uk/observatory/observatorywcc.nsf/0/449B59A1C7151A9E802572CF0038641E/$file/Intro%20&%20CTY.pdf

## Competing interests

The authors declare that they have no competing interests.

## Authors' contributions

KB was the PI, designed the study and secured funding with SAS, TK, SC. NW and SM were employed as researchers to gather the data under the supervision of KB, TC, SC. KB and TC undertook and supervised the statistical analyses, and KB wrote the first and consecutive drafts of the paper with input from the other authors. SM and NW also included some of the data from the overall study in their PhD theses, under the supervision of KB and TC.

## Pre-publication history

The pre-publication history for this paper can be accessed here:

http://www.biomedcentral.com/1472-698X/12/4/prepub
